# Hydrophobic Modification of Chitosan via Reactive Solvent-Free Extrusion

**DOI:** 10.3390/polym13162807

**Published:** 2021-08-21

**Authors:** Tatiana A. Akopova, Tatiana S. Demina, Mukhamed A. Khavpachev, Tatiana N. Popyrina, Andrey V. Grachev, Pavel L. Ivanov, Alexander N. Zelenetskii

**Affiliations:** 1 Enikolopov Institute of Synthetic Polymeric Materials, Russian Academy of Sciences, 70 Profsoyuznaya St., 117393 Moscow, Russia; detans@gmail.com (T.S.D.); muxamed_hav@mail.ru (M.A.K.); tanjapopyrina@yandex.ru (T.N.P.); ivanovpl@inbox.ru (P.L.I.); an-zel@mail.ru (A.N.Z.); 2 Semenov Federal Research Center for Chemical Physics, Russian Academy of Sciences, 4 Kosygina St., 119991 Moscow, Russia; andrgrachyov@yandex.ru

**Keywords:** chitosan alkylation, alkyl glycidyl ethers, solid state organic reactions, mechanochemical synthesis, hydrophobic derivatives

## Abstract

Hydrophobic derivatives of polysaccharides possess an amphiphilic behavior and are widely used as rheological modifiers, selective sorbents, and stabilizers for compositions intended for various applications. In this work, we studied the mechanochemical reactions of chitosan alkylation when interacting with docosylglycidyl and hexadecylglycidyl ethers in the absence of solvents at shear deformation in a pilot twin-screw extruder. The chemical structure and physical properties of the obtained derivatives were characterized by elemental analysis, FT-IR spectroscopy, dynamic light scattering, scanning electron microscopy, and mechanical tests. According to calculations for products soluble in aqueous media, it was possible to introduce about 5–12 hydrophobic fragments per chitosan macromolecule with a degree of polymerization of 500–2000. The length of the carbon chain of the alkyl substituent significantly affects its reactivity under the chosen conditions of mechanochemical synthesis. It was shown that modification disturbs the packing ability of the macromolecules, resulting in an increase of plasticity and drop in the elastic modulus of the film made from the hydrophobically modified chitosan samples.

## 1. Introduction

The hydrophobization of polysaccharides is widely used to change their hydrophilic–lipophilic balance and, accordingly, the rheological properties of their aqueous solutions. Lipophilic interaction of the introduced side substituents leads to aggregation of the macromolecules in an aqueous medium, which results in the formation of nanoparticles with a “core-shell” structure, micelles, or gels, depending on the polymer concentration, temperature, number of substituents, and the length of their alkyl chains [1,2,3,4,5]. Such derivatives of chitosan are used mainly for the development of amphiphilic drug delivery vehicles [6], for cleaning the water surface from oil contamination, including with subsequent regeneration of oils [7], as separating membranes [8], and for stabilizing oil-in-water emulsions [9,10,11]. Current trends in coatings also include the usage of chitosan as polymeric matrices having the intrinsic antimicrobial properties, and thus a preservative ability [12]. The introduction of hydrophobic substituents into chitosan increases its antibacterial activity [13] as well coagulation ability when used as hemostatic agents [14]. A modern trend involving the replacement of non-degradable polymers by natural ones requires the development of advanced approaches to fabricate composite materials. Therefore, another promising application of hydrophobized chitosan is the fabrication of filled polymeric materials based on polyolefins, such as polyethylene, polypropylene, etc. The presence of hydrophobic units within polysaccharide structure could positively affect the distribution of chitosan over polyolefin matrix, which is especially important for processing polymers via additive technologies, in particular for 3D printing where polyolefin-based filaments are successfully used [15,16]. The fabrication of composite filaments for 3D printing usually requires several steps of component mixing, and preliminary modification of their chemical structure could facilitate the compatibility of the filler and polyolefin matrix [17,18].

Hydrophobization is usually carried out by introducing alkyl substituents of various lengths into the structure of chitosan due to reactions of its amino groups with fatty acids or their anhydrides (acylated derivatives) [6,19] or by interaction with aldehydes followed by the reduction of azomethine bonds to secondary amines (alkylated derivatives) [3,7,20,21]. The processes of derivatization of natural polysaccharides are always accompanied by their preliminary activation in order to destroy the highly organized supramolecular structure, preventing the dissolution and melting of polysaccharides without decomposition [22]. These processes require the use of a large excess of organic solvents followed by their expensive regeneration and always begin as heterogeneous reactions in the preparation of hydrophobic derivatives [23,24]. Therefore, organic solid-state reactions based on the joint action of high pressure and shear strains in a solid mixture of reagents, including polymers [25,26,27], have numerous advantages since they are infinitely high-concentration reactions and proceed much more efficiently and faster frequently than solution reactions [28,29,30]. Shear deformation provides numerous possibilities to circumvent many processing obstacles typical to the interaction of hydrophilic polymers and hydrophobic organic reagents [31,32]. So, we developed a mechanochemical approach as an alternative method to produce the hydrophobically modified polysaccharides, allowing to increase the availability of polysaccharide functional groups in the processes of their chemical modification.

The main aim of this work was the study of solvent-free mechanochemical reactions of chitosan alkylation when interacting with docosylglycidyl and hexadecylglycidyl ethers at shear deformation in a pilot twin-screw extruder. The chemical structure and physical properties of the obtained derivatives were characterized by elemental analysis, FT-IR spectroscopy, dynamic light scattering (DLS), scanning electron microscopy (SEM), and mechanical tests of the films cast from their acidic aqueous solution. 

## 2. Materials and Methods 

Crab chitin (moisture 4.3%, ash content 1.8%) was purchased from Xiamen Fine Chemical Import & Export CO., LTD (China). Chitosan (molecular weight (Mw) of 80,000; degree of acetylation (DA) of 0.13) and chitosan (Mw of 140,000; DA of 0.07) were prepared through the mechanochemical alkaline deacetylation of this chitin (ISPM RAS, Russia) (Samples Ch1-LMw and Ch2-LMw, respectively) in accordance with the published procedure [33]. Chitosan (Mw of 350,000; DA of 0.20) was purchased from SONAT (Russia) (Sample Ch-HMw). C16 Glycidil ether (HAGE 16, 2-[(hexadecyloxy)methyl]--oxirane, C19H38O2, MW 298.511, MP 28–31 °C, CAS-No 15965-99-8) and C22 Glycidil ether (HAGE 22, [(docosyloxy)methyl]-oxirane, MP 55–65 °C, CAS-No 20920-10-9) of synthetic grade were purchased from SACHEM Europe B.V. (The Netherlands) and marked as C16 and C22 modifier. All solvents were purchased from Acros Organics (Belgium) as analytical grade and were used without further purification.

The viscosity average molecular weight Mw of chitosan samples was determined by viscometry and calculated by Mark–Kuhn–Houwink equation:

[η] = *K*_m_*M*^a^, (1)

where *K*_m_ = 1.38 × 10^−4^ and a = 0.85 [34]. 

The degree of deacetylation was determined by potentiometry. Hydrodynamic characteristics of the initial chitosan samples were determined with an Ubbelohde viscometer with a capillary diameter of 0.54 mm at 25 °C, and the pH values, with an Ecotest-120 ionomer (NPP Infraspak-Analit, Novosibirsk, Russia) with a combined electrode, and accuracy ± 0.1. Solutions of concentrations с = 0.5 and 1 g dL^−1^ were prepared by dissolving a weighed portion of the chitosan in an acetate buffer (0.33 M CH_3_COOH + 0.2 M CH_3_COONa) with рН 4.4, and in hydrochloric acids of 0.1 M concentration, respectively. The solutions were filtrated through a 0.45-µm syringe filter (Carl Roth, Karlsruhe, Germany) before analysis. The DA of chitosan was calculated as following:

DA = 100 − 203m/G − 161m + 203m,(2)

where 203—Mw of acetylated chitosan unit; m—number of moles of amino-containing units in a weighed sample of chitosan m = V × T; V—volume of NaOH corresponding to neutralization of the protonated form of amino groups of chitosan, ml; Т—titer of NaOH solution in mol/mL; G—weight of chitosan, g; 161—molecular weight of an elementary unit of chitosan. The hydrodynamic properties of chitosan solution are given in Table A1.

Pre-mixing of alkylation reagents with chitosan was conducted as follows. Alkyl glycidyl ethers were dissolved in acetone (1/4 *w*/*v*). Chitosan was soaked in the reagent solution at predetermined ratios of the components at RT for 5 min. The mixture was dried in a vacuum oven without heating for 1 h. The synthesis was carried out in a pilot twin-screw extruder (Berstorff, Germany) with parallel rotation of screws (d = 40 mm) and controlled heating (4 zones). Pre-mixed reagents were fed manually at screw rotation speed of 60 rpm. The processing temperature of 40 and 50 °C for the HAGE 16 and HAGE 22, respectively, was set in all heating zones. With a residence time of ca. 2–3 min, a feed rate of 30 g min^−1^ was achieved. The obtained products were marked as Ch2-L-C22-3, Ch2-L-C22-10, Ch1-L-C16-3, and Ch-H-C16-5 samples, where first indicates initial chitosan type, then modifier type and its weight percentage in the processed mix. Table 1 shows data concerning the conditions for the synthesis of the samples and their main characteristics.

The products were purified with chloroform followed by fractionation by dissolution in 2% CH_3_COOH and separation of soluble and insoluble products by centrifugation at 9500 rpm. Then, the soluble fractions were precipitated with 1 M NaOH. The precipitates and insoluble products were rinsed with distilled water up to neutral pH and freeze-dried to produce the soluble (marked using “s” as suffix) and insoluble (“ins”) fractions in powder form.

Content of carbon, nitrogen, and hydrogen was revealed using FLASH-2000 Organic Elemental Analyzer (Thermo Fisher Scientific, Loughborough, UK). Glucosamine content was calculated from the EA data using C/N ratio for both pure and modified chitosan.

**Figure 1:**
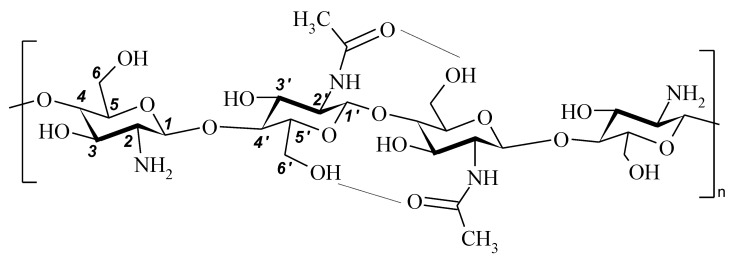
Chemical structure of chitosan.

FT-IR spectra were recorded on a Bruker Vertex 70 spectrometer (USA). All spectra were initially collected in ATR mode at resolution of 4 cm^−1^ by employing an ATR-mono-reflection Gladi ATR (Pike Technologies, Madison, WI, USA) accessory equipped with diamond crystal (*n* = 2.4; angle of incidence 45 deg.). The obtained ATR spectra were converted into IR-Absorbance mode. All the spectra presented in this work were recorded and treated using a set of programs: Bruker Opus (version 6.1). The spectra were normalized using the compound band of stretching vibrations C–O of the pyranose ring at 1075 cm^–1^ as an internal standard [35]. The chemical structure of chitosan and assignments of the polysaccharide main bands are given in Figure 1 and Table 2. The band assignments were made according to [35,36,37,38].

Dynamic light scattering (DLS) of 0.2 wt-% solutions of initial chitosan and soluble fractions of hydrophobic modified (HM) samples in 2%CH_3_COOH was carried out using a Zetatrac particle size analyzer (Microtrac, Inc., Montgomeryville and York, PA, USA). The solutions were filtrated through a 0.45-µm syringe filter (Carl Roth, Karlsruhe, Germany) before analysis. 

Films from chitosan and acid soluble fractions of the HM-samples were fabricated through the casting of 2% respective polymeric solutions in 2% acetic acid on polystyrene Petri dish and drying in a dust-free chamber at RT. The thickness of the films was 90–120 µm. Before mechanical testing, the films were kept in a desiccator at a constant humidity of 81% above the (NH_4_)_2_SO_4_ saturated solution for a week. Mechanical studies of the film samples (4.2 × 10 mm) were carried out using an AGS-H universal testing machine (Shimadzu, Japan) at a speed of 5 mm/min following the guidelines of ASTM D3039/D3039M-08 “Standard test method for tensile properties of polymer matrix composite materials”. Mechanical characteristics of the films, namely tensile strength, elastic modulus, and elongation at break, were calculated as the average value of five measurements of a film sample taking into account its thickness using the instrument software.

Scanning electron microscopy (SEM) of the films was carried out with an aim of PhenomProX (Thermo Scientific, Waltham, MA, USA) operated at 10–15 kV. 

## 3. Results

### 3.1. Structural Characteristics of the Obtained Derivatives

#### 3.1.1. Elemental Analysis

The degree of substitution (DS) of the synthesized *N*-2-hydroxy-3-(hexadecyl) methyl and *N*-2-hydroxy-3-(docosyloxy) methyl derivatives of chitosan was calculated from elemental analysis data from the difference in the molar C/N ratios in the product and in the initial chitosan, referred to the number of carbon atoms in the substituent (see Table 3).

#### 3.1.2. FT-IR Data

**Figure 2:**
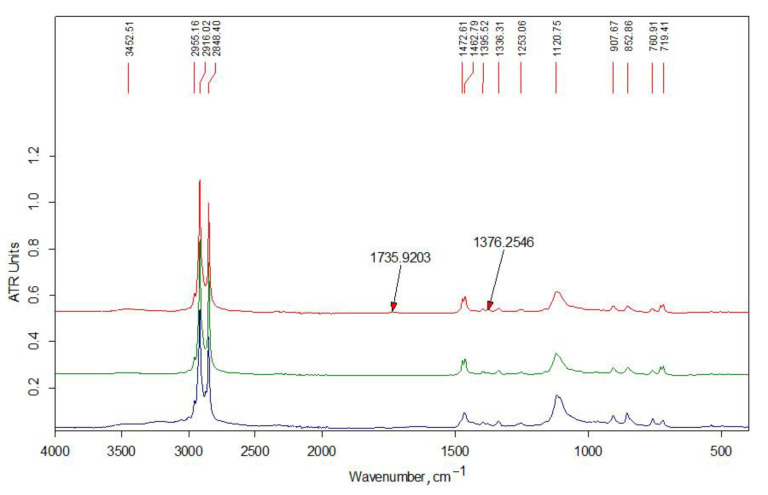
FT-IR spectra (bottom to top): starting reagents HAGE 16 (C16), HAGE 22 (C22); modifier C22 unreacted after co-extrusion.

Figure 2 shows the IR spectra of the initial alkylation reagents and the unreacted modifier extracted from the Ch2-L-C22-10 sample with acetone. All the presented spectra contain an intense doublet of the bands of asymmetric and symmetric stretching vibrations of the methylene groups of the alkyl chain at 2916 and 2848 cm^−1^, respectively, as well as the corresponding bands of their bending vibrations in the low-frequency region of the spectrum. The doublet of the deformation vibrations of the alkyl groups of the C16 modifier at 1472 and 1462 cm^−1^ is the envelope of the bands due to the larger contribution of the –C(H)– and –CH_3_ groups in the relatively short alkyl chain. The bands of stretching vibrations of –C–O– bonds of the glycidyl group are present in the spectra at 1120 and 907 cm^−1^.

**Figure 3:**
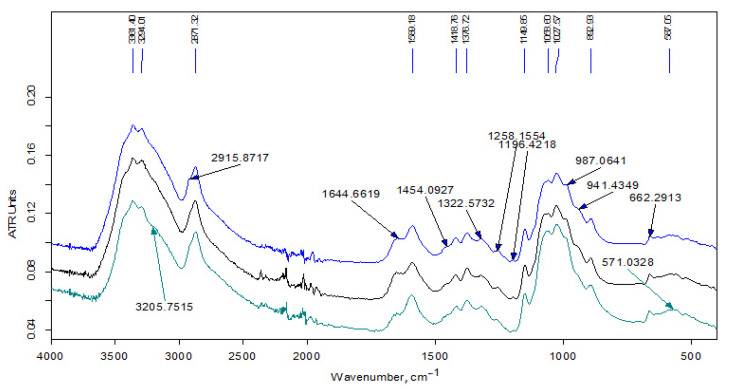
FT-IR spectra of the samples (bottom to top): chitosan Ch2-LMw; chitosan Ch1-LMw; fraction Ch1-L-C16-3s.

Figure 3 shows the IR spectra of the samples of the initial Ch-LMw chitosan and the soluble fraction of the product prepared using hexadecylglycidyl ether (the initial content in the reaction mixture was 3 wt-%). The high-frequency spectral region (3700–2500 cm^−1^) includes a wide band of stretching vibrations of H–O groups, a doublet of overlapping bands of H–C groups (with a maximum at 2871 cm^−1^), as well as a doublet of bands of asymmetric and symmetric stretching vibration NH_2_-groups, which appear in the spectrum as weak bands at 3361 and 3294 cm^−1^ against the background of the band of stretching vibrations of hydroxyl groups.

**Figure 4:**
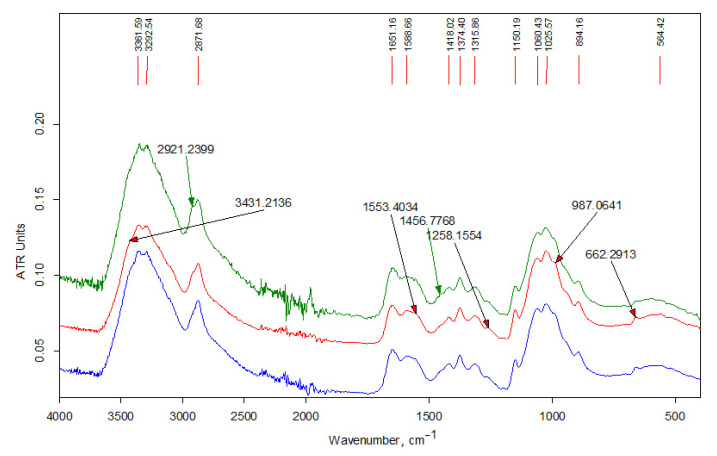
FT-IR spectra of the samples (from bottom to top): chitosan Ch-HMw; fraction Ch-H-C16-5s; fraction Ch-H-C16-5ins.

Figure 4 shows the IR spectra of the initial chitosan Ch-HMw sample as well as soluble and insoluble fractions of its derivative, i.e., Ch-H-C16-5 sample (modifier content 5 wt-%). The spectra of both fractions of this sample also contain an additional absorption band of stretching vibrations of methylene groups, which appears at 2921 cm^−1^ and is better resolved in the spectrum of the fraction of the sample insoluble in an aqueous acidic medium.

**Figure 5:**
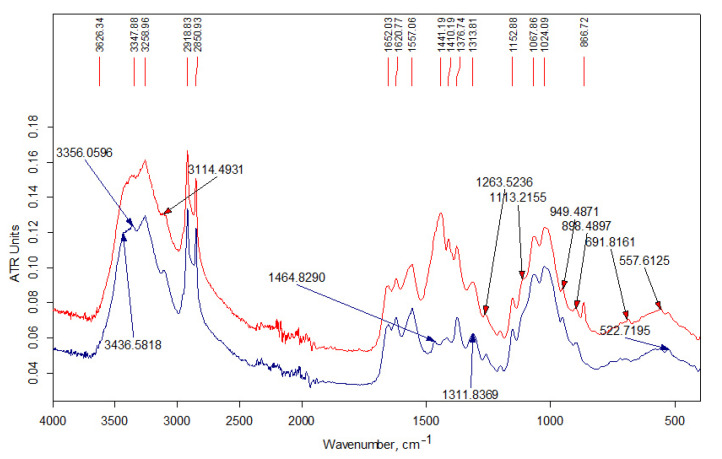
FT-IR spectra of the samples insoluble in acidic aqueous medium: Ch1-L-C16-3ins fraction (bottom); Ch2-L-C22-10ins fraction (top).

Figure 5 shows the IR-spectra of the based on Ch-LMw chitosan fractions insoluble in aqueous media. The intense doublet of the bands of asymmetric and symmetric stretching vibrations of the methylene groups of the alkyl chain at 2918 and 2850 cm^−1^, respectively, present in the spectra, practically repeats the shape and intensity of the absorption bands in alkylation reagents. In the low-frequency region of the spectra, the absorption is determined by deformation vibrations of NH_2_ groups, including those involved in the alkylation reaction, resulting in secondary amine formation.

### 3.2. Behavior in Solution and Properties of the Films

#### 3.2.1. Dynamic Light Scattering

**Figure 6:**
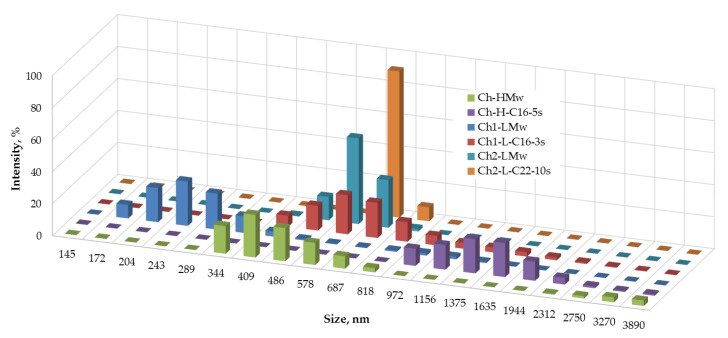
Number-weighted size distribution of macromolecular associates of native chitosan samples (Ch1-LMw, Ch2-LMw and Ch-HMw) and their *N*-alkyl derivatives in 0.2 wt-% solutions in aqueous acetic acid.

**Figure A1:**
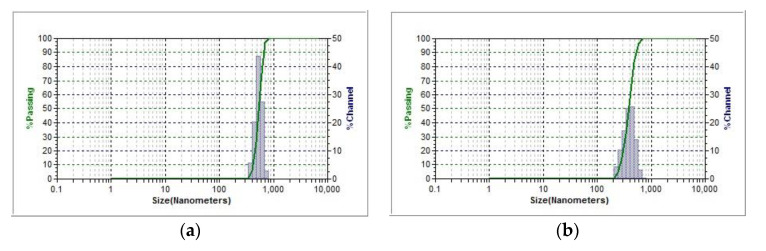
Original histograms of number-weighted size distribution of macromolecular associates of (**a**) N-2-hydroxy-3-(hexadecyl) methyl chitosan (Ch1-L-C16-3s sample, degree of substitution of 0.012) and (**b**) initial chitosan with degree of acetylation of 0.07 and Mw of 140 kDa (Ch2-LMw sample) in 0.2 wt-% solutions in aqueous acetic acid.

The effect of the introduction of the alkyl substituents into the structure of chitosan on its macromolecular behavior in solution was evaluated with the use of DLS. Figure 6 shows that the hydrophobization of both types of chitosan samples led to an increase in the size of associates, while the profile of size distribution was unchanged. Original histograms of the number-weighted size distribution of macromolecular associates of N-2-hydroxy-3-(hexadecyl) methyl chitosan with degree of substitution 0.012 and initial chitosan with degree of acetylation 0.07 and Mw of 140 kDa in 0.2 wt-% solutions in aqueous acetic acid are given in Figure A1.

#### 3.2.2. Mechanical Tests

Mechanical characteristics of the films made of both LMw and HMw chitosan samples and their water-soluble derivatives prepared using C16 modifier are summarized in Table 4. Deformation curves as well as SEM micrographs of the films under investigation are presented in Supplementary Figures S1 and S2, respectively.

## 4. Discussion

**Figure 7:**
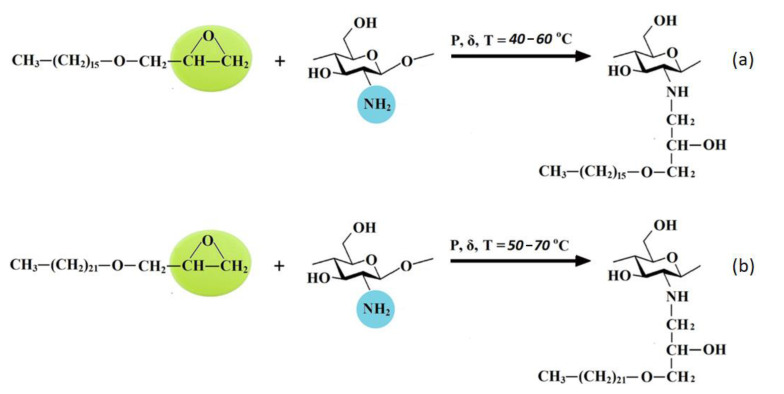
The proposed schemes of chitosan hydrophobization using hexadecylglycidyl (**a**) and docosylglycidyl (**b**) ethers as alkylation agents.

The proposed schemes of interactions of chitosan with HAGE 16 and HAGE 22 are shown in Figure 7a,b, respectively.

The chemical structure of the obtained samples was studied by elemental analysis and IR spectroscopy. The spectrum of the unreacted modifier extracted from the Ch-L-C22-10 sample (see Figure 2) contains traces of acetone (no more than 1–2%). Otherwise, it indicates an insignificant contribution of the side reaction of hydrolysis of the glycidyl group during extrusion. In the spectra of initial LMw chitosan samples (Figure 3), the relative intensity of the bands of the doublet of deformation vibrations of NH_2_ groups at 1590 cm^−1^ and stretching vibrations of C=O in the amide group (Amide-I) at about 1650 cm^−1^ indicate a smaller degree of acetylation of Ch2-LMw, which is consistent with the obtained data of potentiometric titration. 

FTIR analysis of the products are presented in the Figure 3, Figure 4 and Figure 5. In the high-frequency spectral region (3700–2500 cm^−1^), the spectra of water-soluble fractions (Figure 3) contain an additional absorption band (in the form of a shoulder) of stretching vibrations of methylene groups at 2915 cm^−1^, which is related to alkyl substituents in the chitosan structure. The spectra of insoluble fractions of the samples also contain an additional absorption band of stretching vibrations of methylene groups, which appears at 2918–2921 cm^–1^ (Figure 4 and Figure 5). Moreover, these bands are much better resolved. The intense doublet of the bands of asymmetric and symmetric stretching vibrations of the methylene groups of the alkyl chain at 2918 and 2850 cm^−1^, respectively, present in the spectra of insoluble fractions of the LMw chitosan samples (Figure 5), practically repeats the shape and intensity of the absorption bands in the alkylation reagents. All the above indicates a relatively higher DS of chitosan functional groups by alkyl substituents in these fractions. The data of elemental analysis also confirmed this observation (see Table 3, “ins” fractions vs. “s” ones). Moreover, DS of insoluble fractions is approximately the same in all samples, despite the large difference in the content of modifiers in the initial mixtures.

The absorption in the 1650–1500 cm^−1^ region is determined by δNH_2_. It can be seen from the spectra that the DA of the commercial chitosan sample presented in Figure 4 is higher than in the samples obtained by solvent-free extrusion. That also corresponds to the characteristics declared by the manufacturer. The change in the relative intensity of the bands of deformation vibrations of NH_2_ groups due to the alkylation reaction is practically imperceptible, since for secondary amines this band (in the region of 1600–1500 cm^−1^) is usually weak. In addition, the simultaneous presence of OH groups in amine molecules makes it difficult to identify the absorption bands of the amino group [29]. At the same time, a detailed examination of this region in the IR spectra of the samples based on relatively low-molecular-weight chitosan (Figure 5) shows the presence of an intense band at 1557 cm^−1^, which should be attributed to the absorption of a secondary amine. This band is significantly shifted towards lower frequencies due to the presence of a hydroxyl group next to it in the structure of the products (see the interaction scheme in Figure 6). In the spectra of the initial chitosan, this band is weakly expressed and refers to the vibrations of δNH and νCN bonds in the amide group (Amide-II).

Rheological behavior of pristine and hydrophobized polysaccharides is logically changed as a function of their chemical structure. In the case of chitosan, due to its polyelectrolyte nature, the properties of the aqueous solutions also strongly depend on the molecular characteristics of the initial polymer, such as Mw and DA [2]. As follows from the data in the Figure 6, the hydrodynamic volume of the non-modified chitosan samples grows with an increase in their molecular mass. The mean size of Ch1-LMw was 250 nm, whereas for the other two samples a mean size of the associates in range of 400–600 nm was observed. The hydrophobization of chitosan macromolecules led to an increase of the hydrodynamic volume that indicates a domination of the intermolecular associating process in the aqueous system. The size of macromolecular associates of hydrophobic derivatives was also larger in the case of an initial polymer with higher Mw, i.e., Ch-H-C16-5s > Ch1-L-C22-10s > Ch1-L-C16-3s (Figure 6). 

Synthesized hydrophobic chitosan derivatives are targeted to be used as biodegradable fillers within polyolefin-based materials, but it is also possible to use them as substantive polymeric materials. Therefore, their film-forming ability from acetic acid aqueous solution was tested as well. SEM observation of the films of initial chitosan samples and the acid soluble fractions of their HM-samples (Supplementary Figure S2) demonstrates the homogeneity all of the patterns, more pronounced in the case of Ch-HMw-based films. Mechanical tests showed that modification disturbs the packing ability of the macromolecules that results in an increase of plasticity and drop in the elastic modulus of the film of HM-samples (Table 4, Supplementary Figure S1). These changes are favorable to the improvement of the properties of chitosan-based materials in a number of applications, in particular as biopolymeric dressings and coatings [39]. The relatively small degree of substitution in the soluble fractions does not lead to the formation of an entanglement network due to lipophilic interactions. Therefore, the strength of the modified film samples also slightly decreases. 

## 5. Conclusions

In this work, we have shown the efficiency of the solvent-free method, which makes it possible to carry out the chemical modification of polymers in the absence of liquid dispersion media as well as any catalysts to obtain hydrophobic derivatives of chitosan. The solid state reactions of chitosan alkylation when interacting with docosylglycidyl and hexadecylglycidyl ethers at shear deformation in a pilot twin-screw extruder were investigated. According to calculations, the DS of amino groups of chitosan with alkyl substituent for the soluble products was 0.006–0.016, that corresponds to 5–10 hydrophobic moieties per chitosan macromolecule with a degree of polymerization of 500–800 (Ch-LMw samples) and 12 substitutes per macromolecule for Ch-HMw sample with DP of 2000. A significant part of the products (20–30 wt-%) lost the ability to dissolve in acidic aqueous media, which is obviously associated with a sufficiently long length of alkyl substituents. DS in such fractions is logically higher and reaches 0.2. The following conclusions can be drawn from the FTIR analysis. The modifier practically does not undergo the side reaction of hydrolysis of the glycidyl group during solvent-free co-extrusion, as evidenced by the spectrum of the sample obtained by extracting unreacted low molecular weight reagents. The length of the carbon chain of the alkyl substituent significantly affects its reactivity under the chosen conditions of mechanochemical synthesis. Thus, the DS of the samples is practically the same when the content of the C22 modifier is 10 wt-% and the C16 modifier is 3 wt-% in the initial mixtures. Hydrophobic substituents in the structure of the obtained samples led to a loss of solubility in aqueous media, and the DS within them is quite high. Thus, the absorption bands of alkyl substituents and secondary amine are well resolved in the spectra of insoluble fractions of the samples. The spectral data obtained are in good agreement with the data of the calculations based on the chemical analysis. Changes in the plasticity of the modified chitosan films are favorable to the improvement of the properties and the increased use of natural polymers as polymeric coatings. The effect of alkyl fragment presence within the chemical structure of chitosan on its dispersibility within polyolefin matrix will be tested subsequently.

## Figures and Tables

**Table 1:** Conditions for obtaining samples and their characteristics.

Sample Code	Modifier Content, wt-%	Temperature of Treatment ^1^, °C	Solubility in 2% Aqueous CH_3_COOH	Mw, ^2^ kDa	DD, ^3^ %
Ch-HMw	-	-	95	350	80
Ch1-LMw	-	-	94	80	87
Ch2-LMw	-	-	92	140	93
Ch2-L-C22-3	3	50–60	75	–	–
Ch2-L-C22-10	10	50–70	57	–	–
Ch1-L-C16-3	3	40–60	76	–	–
Ch-H-C16-5	5	40–60	72	–	–

^1^ The upper value of the interval was recorded in the second zone, where the power (kneading) elements of the screws are located. ^2^ Average viscosity molecular weight according to capillary viscometry data; ^3^ degrees of deacetylation (glucosamine unit content) according to potentiometric titration data.

**Table 2:** Interpretation of IR spectra.

Wave Number, cm^−1^	Interpretation [35,36,37,38]
3480	νОН in hydrogen bonds of (C6) H_2_OH groups
3450	νОН in hydrogen bonds between O_3_ and O_2_ atoms
3370	νNН asymmetrical
3295	νNН symmetrical
3265	νNН (Amid II band)
3100	combined band νNН
2950	νCН_3_ asymmetrical
2930	νCН_2_ asymmetrical
2890–2880	νCН_3_, νCН_2_ symmetrical, νCН in the ring
3000–2840	νCН in alkyl substituents
1660	νC=О (пoлoса амид I) in hydrogen bonds C=О---НN
1630	νC=О (пoлoса амид I) in hydrogen bonds with О_3_
1600	δNН_2_
1560	δNН (Amid II band), νCN
1475–1450	δCН_2_ in alkyl substituents
1470–1430	δCН_3_ in alkyl substituents
1430–1420	δCН, δCН_2_, δCН_3_ in chitin and chitosan
1395–1365	δCН_3_ in alkyl substituents
1390	νCН, δCН symmetrical
1380	δCН_3_ symmetrical
1375	νCN, δCН_2_
1320	δNН, δCН_2_ symmetrical
1310	δNН (Amid III band), δCН_2_ symmetrical
1260–1200	in (C_3_)НОН groups
1155	νCО asymmetrical of acetal bond
1110	νCО asymmetrical in the ring
1070–1030	νCО in the ring, in (C_3_)НОН and (C_6_)Н_2_ОН groups
975	C-CН_3_
895	νCО asymmetrical, δCН
770–720	δCН_2_ in alkyl substituents

**Table 3:** Elemental analysis data for the initial chitosan samples and the products of their interaction with alkylation agents.

Sample	%C	%Н	%N	C/N	DS
Ch-HMw	43.74 ± 0.14	6.79 ± 0.11	7.82 ± 0.02	6.53	–
Ch1-LMw	43.62 ± 0.12	6.67 ± 0.12	7.93 ± 0.02	6.42	–
Ch2-LMw	43.03 ± 0.21	6.82 ± 0.04	8.17 ± 0.03	6.23	–
Ch1-L-C16-3s	40.85 ± 0.10	7.85 ± 0.08	7.21 ± 0.01	6.61	0.012
Ch1-L-C16-3ins	43.56 ± 0.01	7.05 ± 0.01	5.33 ± 0.01	9.53	0.19
Ch-H-C16-5s	40.85 ± 0.10	7.70 ± 0.10	7.20 ± 0.01	6.62	0.006
Ch-H-C16-5ins	43.56 ± 0.01	7.05 ± 0.01	5.32 ± 0.01	9.55	0.19
Ch2-L-C22-3s	40.15 ± 0.08	6.65 ± 0.12	7.84 ± 0.02	5.98	–
Ch2-L-C22-3ins	43.58 ± 0.01	6.79 ± 0.11	6.28 ± 0.02	8.10	0.085
Ch2-L-C22-10s	40.72 ± 0.09	7.30 ± 0.10	7.21 ± 0.02	6.59	0.016
Ch2-L-C22-10ins	45.90 ± 0.10	8.31 ± 0.08	5.6 ± 0.01	9.56	0.15

**Table 4:** Mechanical properties of the films of initial and the modified chitosan.

Sample	Tensile Strength, MPa	Elongation at Break, %	Elastic Modulus, MPa
Ch-HMw	71 ± 2.3	19 ± 2.1	1650 ± 35
Ch-H-C16-5s	65 ± 1.5	23 ± 1.4	750 ± 18
Ch1-LMw	37 ± 1.3	4 ± 3.3	1623 ± 32
Ch1-L-C16-3s	30 ± 1.1	8 ± 2.1	877 ± 16

## Data Availability

The data presented in this study are available on request from the corresponding author.

## Supplementary Materials

The following are available online at https://www.mdpi.com/article/10.3390/polym13162807/s1, Figure S1: Deformation curves of the film samples of initial and the modified chitosan, Figure S2: SEM micrographs of the films made of: (a) Ch1-LMw sample (DP of 500, DA of 0.13); (b) Ch1-L-C16-3s (average 6 alkyl substitutes per chitosan macromolecule); (c) Ch-HMw sample (DP of 2000, DA of 0.2); (d) Ch-H-C16-5s (average 12 alkyl substitutes per chitosan macromolecule).

## Appendix A

**Table A1:** Hydrodynamic properties of Ch2-LMw chitosan solution.

C, ^1^ g dl^−1^	t, sec	η_rel_	η_sp_	η_sp_/C
0.45	363	3.42	2.42	5.35
0.33	276	2.60	1.60	4.82
0.26	230	2.17	1.17	4.46
0.22	197	1.86	0.86	4.2

**^1^** C—polymer concentration in solution; t—the solution flow time; η_rel_—relative viscosity (ratio of solution flow time to solvent flow time); η_sp_—specific viscosity (ratio of the difference between the viscosities of the solution and the solvent to the viscosity of the solvent).

Intrinsic viscosity was determined by graphical extrapolation of η_sp_/C values obtained for several concentrations to zero concentration. According to the data and calculations, it was found that intrinsic viscosity was 3.26 dl g^−1^, and Mw of the chitosan sample was 140 kDa.
