# Supplementary material for: Hydrophobic Modification of Chitosan via Reactive Solvent-Free Extrusion
Source: Polymers (Basel). 2021 Aug 21;13(16):2807. doi: 10.3390/polym13162807 (PMC8399264; doi:10.3390/polym13162807)
Supplement: Supplementary file 1 [file polymers-13-02807-s001.zip › Figure S2.pdf]

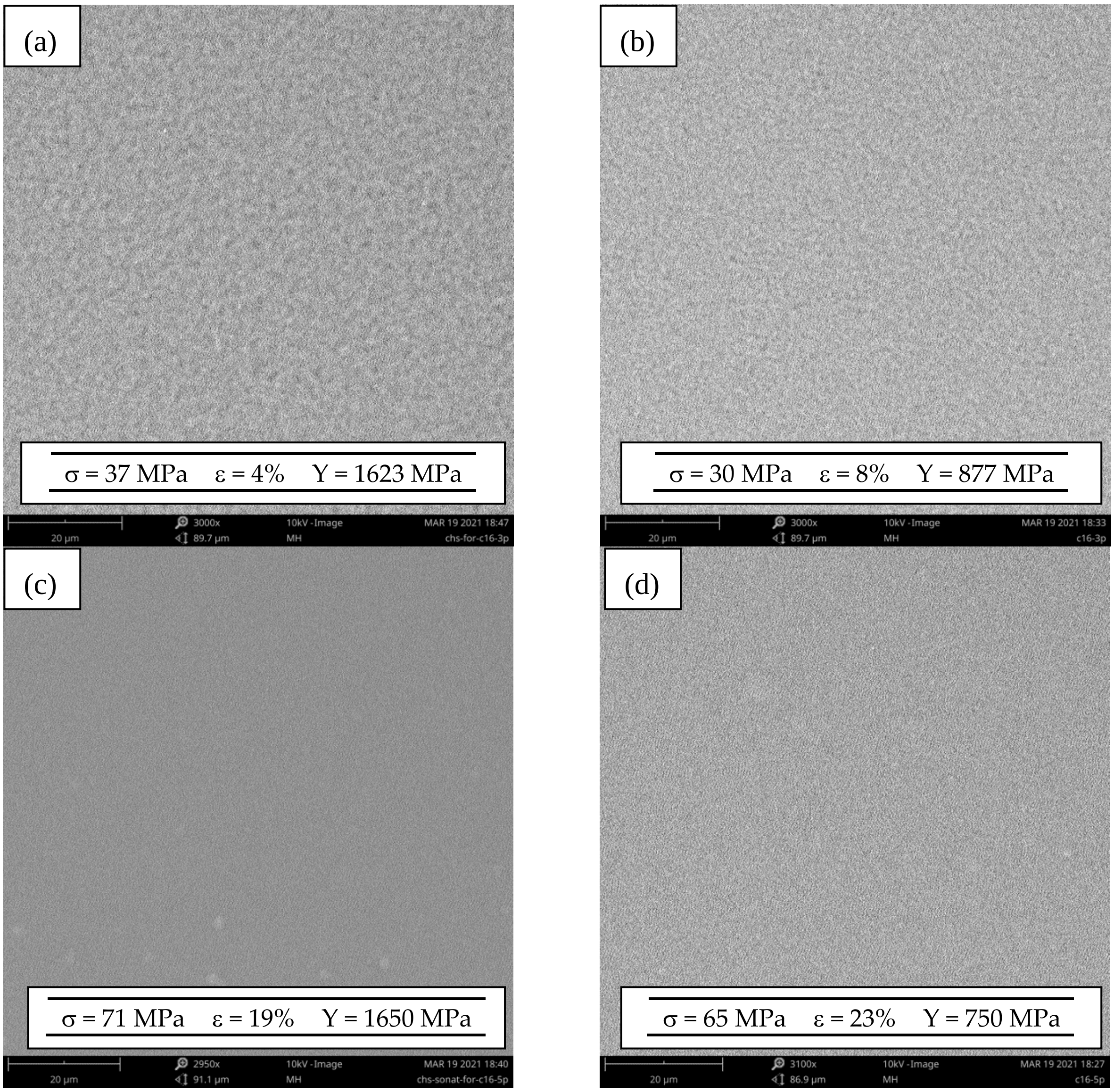

Figure S2. SEM micrographs of the films made of: (a) Ch1-LMw sample (degree of polymerization (DP) of 500, degree of acetylation (DA) of 0.13); (b) Ch1-L-C16-3s (fraction soluble in AcOH, average 6 alkyl substitutes per chitosan macromolecule); (c) Ch-HMw sample (DP of 2000, DA of 0.2); (d) Ch-H-C16-5s (fraction soluble in AcOH, average 12 alkyl substitutes per chitosan macromolecule).
