# Supplementary figures and images for: Hydrophobic Modification of Chitosan via Reactive Solvent-Free Extrusion
Source: Polymers (Basel). 2021 Aug 21;13(16):2807. doi: 10.3390/polym13162807 (PMC8399264; doi:10.3390/polym13162807)

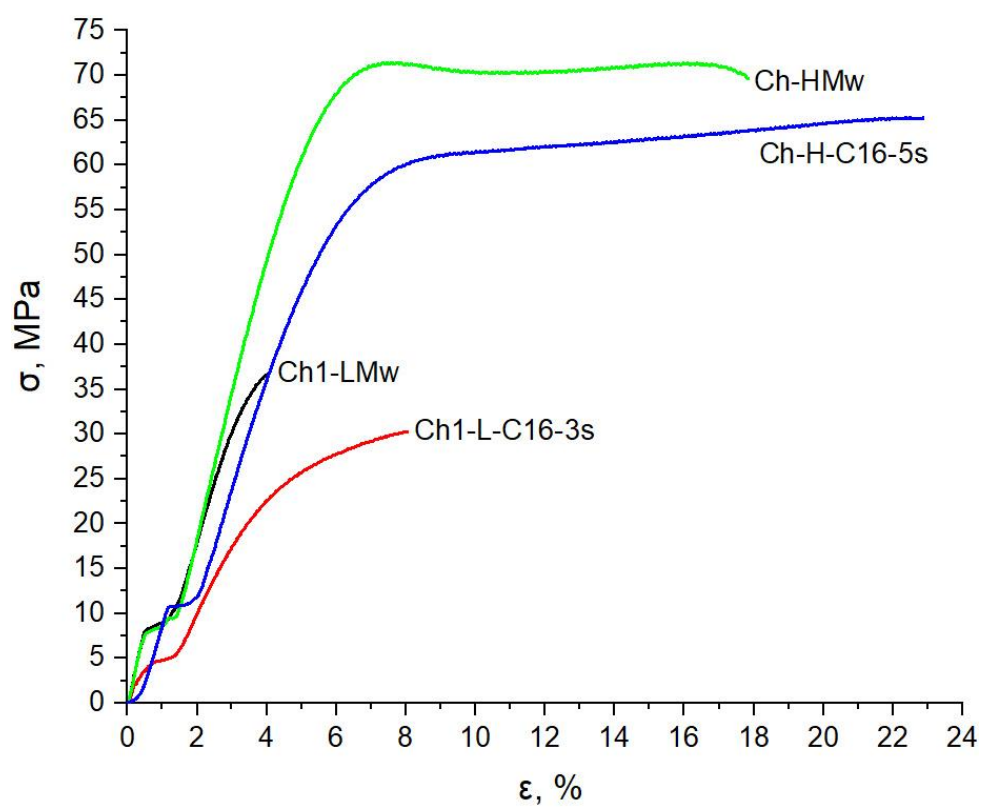

**Figure S1.** Deformation curves of the film samples of initial and the modified chitosan.

Supplement: Supplementary file 1 [file polymers-13-02807-s001.zip › Figure S1.pdf]
